# Factors associated with workplace violence against Chinese healthcare workers: an online cross-sectional survey

**DOI:** 10.3389/fpubh.2024.1295975

**Published:** 2024-03-14

**Authors:** Yu Xiao, Ting-ting Chen, Shao-yi Zhu, Chun-ya Li, Ling Zong

**Affiliations:** ^1^Psychosomatic Medical Center, The Fourth People’s Hospital of Chengdu, Chengdu, China; ^2^Psychosomatic Medical Center, The Clinical Hospital of Chengdu Brain Science Institute, MOE Key Lab for Neuroinformation, University of Electronic Science and Technology of China, Chengdu, China; ^3^Nursing Department, West China Hospital of Sichuan University, Chengdu, China; ^4^Department of Psychiatry, Shantou University Mental Health Center, Shantou, China; ^5^Department of Judicial Expertise, Zhongshan Third People’s Hospital, Zhongshan, China

**Keywords:** career satisfaction, China, healthcare system, healthcare workers, university hospital, occupational safety, workplace violence

## Abstract

**Objectives:**

Workplace violence (WPV) against healthcare workers (HCWs) has reached significant levels globally, impeding the quality and accessibility of healthcare systems. However, there is limited available knowledge regarding the determinants linked with WPV among HCWs and the discrepancies observed across various levels of hospitals in China. The objective of the present research was to investigate the factors linked to WPV and job satisfaction among HCWs in China.

**Methods:**

A self-developed questionnaire based on WeChat was employed to collect data. The questionnaire consisted of demographic information as well as occupational factors. To measure WPV, the Chinese version of the Workplace Violence Scale was utilized. Career satisfaction was assessed through two questions regarding career choices. The collected data was analyzed using descriptive analyses, chi-square tests, and multivariate logistic regressions.

**Results:**

A total of 3,781 valid questionnaires (1,029 doctors and 2,752 nurses) were collected. Among all participants, 2,201 (58.2%) reported experiencing at least one form of WPV in the past year, with emotional abuse being the most frequent occurrence (49.7%), followed by threats (27.9%). The multivariate logistic regression analysis revealed several risk factors associated with WPV, including male gender, shift work, senior professional title, bachelor’s degree education, employment in secondary-level hospitals, and working over 50 h per week (*p* < 0.05). Career satisfaction among HCWs who experienced high levels of WPV was low, with only 11.2% remaining confident in their profession, and a mere 2.0% supporting their children pursuing careers in healthcare.

**Conclusion:**

WPV poses a significant challenge within the Chinese healthcare system. Efforts should be made to address the identified risk factors and promote a safe and satisfying working environment for HCWs.

## 1 Introduction

Workplace violence (WPV) is a serious issue that has been defined by the World Health Organization (WHO) as incidents where staff are subjected to abuse, threats, or assault in connection to their work, including during their commute to and from work, and that directly or indirectly challenge their health, safety, or well-being (1). WPV can be classified into distinct categories: physical assault (PA), which encompasses acts involving physical contact such as biting or beating; emotional abuse (EA), defined by mistreatment through the use of words, such as cursing; threats (T), which involve the use of written, verbal, or physical force to induce fear of negative consequences; verbal sexual harassment (VSH), characterized by unwelcome remarks of a sexual nature; and sexual abuse (SA), encompassing unwanted touching or any other form of unwelcome sexual behaviors.

Violence in the healthcare sector is a growing problem with significant implications for workplace safety (2). Healthcare workers (HCWs) are more vulnerable to violence compared to those in other professions (3, 4). Acts of violence against medical care providers have detrimental effects on their psychological well-being, productivity, and trust in management and colleagues (2, 5). A previous study (6) indicated that WPV was negatively correlated with job satisfaction (*r* = −0.228, *p* < 0.01). Once HCWs experience WPV, their negative emotions increase, job satisfaction decreases, and it may even lead to resignation (6). Furthermore, such violence can signal underlying tensions between HCWs and patients, which may compromise the accessibility and quality of healthcare services (7). Numerous investigations have been conducted to assess the extent of WPV against HCWs on a global scale (8, 9). For instance, a comprehensive analysis that uncovered a startling truth - every year, one out of every five HCWs across the globe encounters instances of physical violence inflicted by patients or their visitors (10). In the United States, healthcare settings account for 70–74% of workplace assaults (11). The 2019 UK National Health Service (NHS) staff survey reported that 15% of NHS members experienced at least one incident of physical violence from patients, relatives, or the general public within the previous year (12). In Germany, 23% of primary care physicians have encountered severe aggression or violence (13). In Iran, the occurrence rate of physical or verbal WPV against emergency medical services personnel stands at 36 and 73%, respectively (14). A study conducted among nurses in South Korea found that 74.3% of respondents had experienced verbal abuse in the past 3 months (15). The COVID-19 pandemic has exacerbated physical and verbal abuse toward HCWs, with patients and their families being identified as the main perpetrators (2, 9, 16).

In Chinese mainland, there were approximately 4.08 million doctors and 4.71 million nurses as per the 2021 national health yearbook (17). According to a meta-analysis study, the prevalence of WPV against HCWs in China was found to be 62.4%. The study further revealed rates of verbal abuse, psychological violence, physical violence, sexual harassment, and threatening behaviors at 61.2, 50.8, 13.7, 6.3, and 39.4%, respectively (18). Male HCWs faced elevated levels of WPV in comparison to their female counterparts, as documented in study (19). In addition, inexperienced nurses and those at the graduate level exhibited increased susceptibility to such incidents (20). Furthermore, healthcare employees who work in shifts were found to be at a higher risk of WPV when compared to those who work regular hours (21). Nevertheless, there has been a predominant emphasis in previous research on township or tertiary-level hospitals, as well as specific healthcare specialties (6, 21, 22). Few studies have compared the prevalence and related determinants of different forms of WPV among doctors and nurses across the three levels of hospitals in China (19). Therefore, the aim of this study was to examine the occurrence and distribution patterns of different types of WPV based on demographic and occupational characteristics. Additionally, we sought to identify factors associated with different types of WPV and assess the effect of WPV on professional satisfaction.

## 2 Methods

### 2.1 Participants and procedure

Our study aimed to examine the experiences and perceptions of HCWs in China through a cross-sectional, online e-survey conducted between 15 January 2023 and 20 February 2023. The snowball sampling method was employed to gather data from a diverse range of HCWs. To initiate the survey, a carefully designed and anonymous questionnaire was created on WeChat, a popular social media platform in China. Initially, a group of 30 nurses and 20 doctors from our university hospital, referred to as “original deliverers,” were selected to participate in the study. These individuals were considered key informants who would help disseminate the survey among their networks.

The “original deliverers” received a link to the questionnaire via WeChat, along with a clear introduction about the study. They were given the option to either agree and proceed with the survey or decline and withdraw from participation. It should be noted that completing the survey questionnaire implied voluntary consent to be part of the study. Additionally, the online survey also extended its invitation to the peers and fellow students of the “original deliverers,” encouraging them to actively participate and distribute the questionnaire among their connections, without receiving any kind of remuneration. The survey links were also shared among the respondents’ acquaintances and within relevant WeChat communities. By adopting this methodology, the sample size was expanded, enabling a more comprehensive representation of China’s frontline HCWs across various departments within medical institutions. Throughout the entire process, strict measures were taken to ensure the security and confidentiality of the participants’ data. Anonymity was maintained to safeguard the privacy of the HCWs and encourage honest responses.

### 2.2 Measures

The survey encompassed a range of sociodemographic information, including age, gender, marital status, educational attainment, and occupational particulars like hospital care level, department of work, occupation, professional title, work schedule (shift or non-shift), years of experience, and weekly work hours. Additionally, respondents were given the opportunity to answer two questions regarding their career choice: “Knowing all the risks involved, would you still have opted for a career in the medical field?” and “Do you desire for your child to pursue a career in the healthcare industry?” Similar questions assessing career satisfaction have been utilized in previous studies focusing on WPV (23). These questions have been found to be effective in capturing HCWs’ perceptions and sentiments regarding their career choices, which are directly relevant to understanding WPV dynamics (24, 25).

To gage the frequency of WPV targeting HCWs in China over the preceding 12 months, the Chinese version of the Workplace Violence Scale (WVS) was employed. The WVS has been demonstrated to possess favorable validity and reliability among HCWs in China, with a Cronbach’s coefficient of 0.75 (26). The scale encompasses five dimensions: EA, PA, VSH, T, and SA (26). The frequency of respondents’ exposure to WPV within the past year is assessed by scoring each item on a scale of 0 to 3. The scores correspond to different levels of exposure: 0 indicates no exposure, 1 indicates exposure once, 2 indicates exposure two or three times, and 3 indicates exposure more than three times. The total score is derived by summing the grades from each item, resulting in a range from 0 to 15. Based on the grades, the level of violence experienced is categorized into four groups (none = 0, low = 1 ~ 5, intermediate = 6 ~ 10, high = 11 ~ 15). The survey presented precise definitions for every category of violence, with an aim to offer clarity and accuracy for understanding. Additional information on the survey instrument utilized in this research is available in Supplementary File 1.

### 2.3 Statistical analyses

In this study, the occurrence of any form of WPV among the respondents was analyzed using a binary response (yes/no) code. The demographic and occupational data were summarized using numbers and percentages, along with the rates of five different types of WPV. To identify significant predictors for each type of WPV, multivariate logistic regression analyses were conducted. Factors that showed a *p*-value of 0.1 or less in the chi-square test were included as independent variables in the models. Additionally, the association between the level of WPV and career choice was assessed using the chi-square test. A *p*-value <0.05 was regarded as statistically significant in this study. All statistical analyses were performed using Microsoft Excel 2019 and SPSS 22.0.

## 3 Results

### 3.1 Characteristics of the respondents

The questionnaire was responded to by a total of 3,812 HCWs from all provinces in mainland China in this investigation. Due to incomplete data, 31 participants were excluded. The final analysis included 3,781 respondents, with 72.8% being nurses and 27.2% being physicians. Among the participants, 64.2% were married while 35.8% were single. The majority of respondents (83.2%) were female. In terms of workplace, 71.0% of the participants worked in tertiary-level hospitals. Furthermore, 60.2% held a primary professional title, and 74.6% reported being shift workers. The age distribution of the participants spanned from 19 to 70 years, with a significant proportion (49.1%) falling under the age of 30. The mean age of the participants was 31.4 ± 8.1 years. The demographic and occupational characteristics distribution of the participants can be found in Tables 1, 2.

**Table 1:** The distribution of five types of WPV across various demographic characteristics.

Demographic variables	*n* = 3,781 N (%)	PA	EA	T	VSH	SA
		N (%)	N (%)	N (%)	N (%)	N (%)
Gender						
Male	635 (16.8)	145 (22.8)	374 (58.9)	237 (37.3)	134 (21.1)	91 (14.3)
Female	3,146 (83.2)	468 (14.9)	1,506 (47.9)	817 (26.0)	478 (15.2)	205 (6.5)
*p* value		**< 0.01**	**< 0.01**	**< 0.01**	**< 0.01**	**< 0.01**
Age (years)						
< 30	1858 (49.1)	318 (17.1)	938 (50.5)	425 (22.9)	271 (14.6)	130 (7.0)
30 ~ 39	1,322 (35.0)	198 (15.0)	660 (48.9)	426 (32.2)	215 (16.3)	118 (8.9)
≥ 40	601 (15.9)	97 (16.1)	282 (46.9)	203 (33.8)	126 (21.0)	48 (8.0)
*P* value		0.27	0.31	**< 0.01**	**< 0.01**	0.14
Education level						
Master’s degree or above	473 (12.5)	51 (10.8)	191 (40.4)	132 (27.9)	76 (16.1)	22 (4.7)
Bachelor’s degree	2,567 (67.9)	466 (18.2)	1,344 (52.4)	740 (28.8)	430 (16.8)	217 (8.5)
Associate’s degree or below	741 (19.6)	96 (13.0)	345 (46.6)	182 (24.6)	106 (14.3)	57 (7.7)
*P* value		**< 0.01**	**< 0.01**	0.07	0.28	**0.02**
Marital status						
Married	2,427 (64.2)	378 (15.6)	1,180 (48.6)	750 (30.9)	392 (16.2)	185 (7.6)
Unmarried	1,354 (35.8)	235 (17.3)	700 (51.7)	304 (22.5)	220 (16.2)	111 (8.2)
*P* value		0.15	0.07	**< 0.01**	0.94	0.53

PA, physical assault; EA, emotional abuse; T, threat; VSH, verbal sexual harassment; SA, sexual abuse.

**Table 2:** The distribution of five types of WPV across various occupational characteristics.

Occupational variables	*n* = 3,781 N (%)	PA	EA	T	VSH	SA
		N (%)	N (%)	N (%)	N (%)	N (%)
Level of hospital						
Primary hospital	69 (1.8)	4 (5.8)	22 (31.9)	7 (10.1)	7 (10.1)	3 (4.3)
Secondary hospital	1,028 (27.2)	208 (20.2)	577 (56.1)	314 (30.5)	171 (16.6)	92 (8.9)
Tertiary hospital	2,684 (71.0)	401 (14.9)	1,281 (47.7)	733 (27.3)	434 (16.2)	201 (7.5)
*p* value		**< 0.01**	**< 0.01**	**< 0.01**	0.37	0.18
Department						
Emergency department	445 (11.8)	217 (48.8)	287 (64.5)	201 (45.2)	139 (31.2)	92 (20.7)
Mental health	344 (9.1)	65 (18.9)	226 (65.7)	148 (43.0)	79 (23.0)	35 (10.2)
Pediatrics	236 (6.2)	69 (29.2)	105 (44.5)	59 (25.0)	23 (9.7)	18 (7.6)
Gynecology and obstetrics	232 (6.1)	20 (8.6)	98 (42.2)	51 (22.0)	27 (11.6)	10 (4.3)
Intensive care unit	159 (4.2)	15 (9.4)	89 (56.0)	51 (32.1)	26 (16.3)	4 (2.5)
Internal medicine	1,005 (26.6)	104 (10.3)	513 (51.0)	262 (26.1)	130 (12.9)	63 (6.3)
Surgical department	782 (20.7)	87 (11.1)	398 (50.9)	190 (24.3)	126 (16.1)	51 (6.5)
Operating room	125 (3.3)	5 (4.0)	21 (16.8)	17 (13.6)	12 (9.6)	4 (3.2)
General practice	140 (3.7)	12 (8.6)	40 (28.6)	21 (15.0)	9 (6.4)	4 (2.8)
Diagnosis and subsidiary	313 (8.3)	19 (6.1)	103 (32.9)	54 (17.3)	41 (13.1)	15 (4.8)
*p* value		**< 0.01**	**< 0.01**	**< 0.01**	**< 0.01**	**< 0.01**
Profession						
Doctor	1,029 (27.2)	165 (16.0)	524 (50.9)	334 (32.5)	198 (19.2)	69 (6.7)
Nurse	2,752 (72.8)	448 (16.3)	1,356 (49.3)	720 (26.2)	414 (15.0)	227 (8.2)
*p* value		0.86	0.37	**< 0.01**	**< 0.01**	0.10
Professional title						
Primary	2,276 (60.2)	368 (16.2)	1,130 (49.6)	547 (24.0)	328 (14.4)	184 (8.1)
Intermediate	1,062 (28.1)	168 (15.8)	523 (49.2)	347 (32.7)	170 (16.0)	76 (7.2)
Senior	443 (11.7)	77 (17.4)	227 (51.2)	160 (36.1)	114 (25.7)	36 (8.1)
*p* value		0.75	0.82	**< 0.01**	**< 0.01**	0.63
Years of experience						
< 5	1,448 (38.3)	244 (16.9)	706 (48.8)	311 (21.5)	205 (14.2)	120 (8.3)
6 ~ 10	1,070 (28.3)	151 (14.1)	543 (50.7)	309 (28.9)	149 (13.9)	75 (7.0)
11 ~ 20	805 (21.3)	141 (17.5)	422 (52.4)	282 (35.0)	165 (20.5)	73 (9.1)
> 20	458 (12.1)	77 (16.8)	209 (45.6)	152 (33.2)	93 (20.3)	28 (6.1)
*p* value		0.17	0.09	**< 0.01**	**< 0.01**	0.17
Work schedule						
Shift	2,820 (74.6)	507 (18.0)	1,508 (53.5)	814 (28.9)	480 (17.0)	248 (8.8)
Non-shift	961 (25.4)	106 (11.0)	372 (38.7)	240 (25.0)	132 (13.7)	48 (5.0)
*p* value		**< 0.01**	**< 0.01**	**0.02**	**0.02**	**< 0.01**
Weekly working hours						
> 50 h	574 (15.2)	103 (17.9)	352 (61.3)	183 (31.9)	127 (22.1)	48 (8.4)
40 ~ 50 h	2,238 (59.2)	338 (15.1)	1,108 (49.5)	604 (27.0)	329 (14.7)	168 (7.5)
< 40 h	969 (25.6)	172 (17.7)	420 (43.3)	267 (27.6)	154 (15.9)	80 (8.3)
*p* value		0.08	**< 0.01**	0.06	**< 0.01**	0.67

### 3.2 Incidence and characteristics of medical WPV

The prevalence of WPV among HCWs was found to be 58.2% (2,201/3781). When examining the different types of WPV, it was observed that EA had the highest prevalence rate of 49.7%, followed by T at 27.9%, PA at 16.2%, VSH at 16.2%, and SA at 7.8%. There were significant differences in the one-year prevalence of these different types of WPV based on demographic and occupational characteristics (refer to Tables 1, 2). Upon analyzing the data, it was found that male HCWs exhibited a higher prevalence of PA (22.8% vs. 14.9%, *χ*^2^ = 24.6, *p* < 0.01), EA (58.9% vs. 47.9%, *χ*^2^ = 25.7, *p* < 0.01), T (37.3% vs. 26.0%, *χ*^2^ = 33.9, *p* < 0.01), VSH (21.1% vs. 15.2%, *χ*^2^ = 13.6, *p* < 0.01), and SA (14.3% vs. 6.5%, *χ*^2^ = 44.7, *p* < 0.01) compared to their female counterparts. Furthermore, HCWs with a Bachelor’s degree exhibited the highest rate of PA (18.2%, *χ*^2^ = 23.2, *p* < 0.01), EA (52.4%, *χ*^2^ = 26.6, *p* < 0.01), and SA (8.5%, *χ*^2^ = 8.0, *p* = 0.02) when compared to individuals from other educational backgrounds. In terms of the level of hospital care, HCWs in secondary hospitals were more susceptible to all five types of WPV, with prevalence rates of EA, T, PA, VSH, and SA at 56.1, 30.5, 20.2, 16.6, and 8.9%, respectively. HCWs in emergency department were identified as the most vulnerable group, experiencing high prevalence rates of all five types of violence (EA, PA, T, VSH, and SA were 64.5, 48.8, 45.2, 31.2, and 20.7%, respectively). They were followed by HCWs in mental health departments, pediatric departments, obstetrics and gynecology departments, in terms of vulnerability to WPV. Shift workers demonstrated a higher vulnerability to PA (18.0% vs. 11.0%, *χ*^2^ = 25.5, *p* < 0.01), EA (53.5% vs. 38.7%, *χ*^2^ = 62.5, *p* < 0.01), T (28.9% vs. 25.0%, *χ*^2^ = 5.4, *p* = 0.02), VSH (17.0% vs. 13.7%, *χ*^2^ = 5.7, *p* = 0.02), and SA (8.8% vs. 5.0%, *χ*^2^ = 14.3, *p* < 0.01) compared to non-shift workers. Moreover, HCWs who were engaged in work for over 50 h per week exhibited a higher vulnerability to EA (61.3%, *χ*^2^ = 46.7, *p* < 0.01), and VSH (22.1%, *χ2* = 18.7, *p* < 0.01).

### 3.3 Career choice and the level of WPV

The association between levels of WPV and career choice is presented in Table 3. Among the respondents, a total of 835 (22.1%) said that they would still engage in the medical industry even if they knew the potential risks. In contrast, an overwhelming majority of 91.7% (3,467/3781) expressed their opposition to the idea of their children pursuing a career in the healthcare industry. Notably, HCWs who had experienced higher levels of WPV demonstrated a significantly lower likelihood (*p* < 0.01) to answer affirmatively to the aforementioned questions. Specifically, out of the HCWs who had encountered high levels of WPV (*n* = 98) within the last year, only 11 individuals (11.2%) expressed their intentions to continue pursuing a career in the healthcare sector, whereas a mere 2 individuals (2.0%) would endorse their children pursuing a career in healthcare.

**Table 3:** Relationship between the level of WPV and career choice.

WPV level	(*n* = 3,781) N (%)	Would you still choose to work in the healthcare industry? (%)		Do you support children becoming healthcare professionals? (%)	
		Yes (22.1)	No (77.9)	Yes (8.3)	No (91.7)
High	98 (2.6)	11 (11.2)	87 (88.8)	2 (2.0)	96 (98.0)
Intermediate	351 (9.3)	49 (14.0)	302 (86.0)	17 (4.8)	334 (95.2)
Low	1781 (47.1)	316 (17.7)	1,465 (82.3)	106 (6.0)	1,675 (94.0)
None	1,551 (41.0)	460 (29.7)	1,091 (70.3)	190 (12.3)	1,361 (87.7)
*p* value		**< 0.01**		**< 0.01**	

### 3.4 Factors associated with WPV

The results of the multivariate logistic regression analyses, which examined the factors related to the five categories of WPV, are displayed in Table 4. It was observed that both male gender and working in shifts were identified as significant factors associated with all five categories of medical WPV (*p* < 0.01). HCWs with a bachelor’s degree education were found to be significantly associated with EA (*p* < 0.01), PA (*p* < 0.01), and SA (*p* < 0.01). Additionally, working in secondary hospitals was found to be the strongest correlate for EA (*p* < 0.01), PA (*p* < 0.01), and T (*p* < 0.01), with odds ratios of 2.64 (95% CI: 1.81–3.84), 3.70 (95% CI: 1.97–7.64), and 3.29 (95% CI: 1.95–5.63), respectively. Furthermore, HCWs holding a senior professional title were found to be significantly associated with VSH (*p* < 0.01) and T (*p* < 0.01). The analysis also revealed that engaging in work for more than 50 h per week displayed a significant correlation with the occurrence of EA [OR = 1.71 (95% CI: 1.37–2.12), *p* < 0.01].

**Table 4:** Multivariate logistic regression for the correlation between demographic and occupational factors and five distinct types of WPV.

Risk factors	PA	EA	T	VSH	SA
	OR (95% CI)	OR (95% CI)	OR (95% CI)	OR (95% CI)	OR (95% CI)
Male	1.76(1.24–2.33)^a^	1.52(1.17–1.84)^a^	1.67(1.43–2.02)^a^	1.71(1.36–2.15)^a^	2.13(1.61–3.04)^a^
Shift work	1.62(1.31–2.06)^a^	1.66(1.44–1.98)^a^	1.40(1.19–1.73)^a^	1.43(1.12–1.81)^a^	1.95(1.58–2.67)^a^
Education level					
Master’s degree and above	Ref				
Associate’s degree and below	1.17(0.83–1.62)^b^	1.12(0.86–1.37)^b^	-	-	1.54(1.12–2.29)^a^
Bachelor’s degree	1.81(1.20–2.54)^a^	1.25(1.08–1.51)^a^	-	-	1.90(1.25–2.76)^a^
Level of hospital					
Primary	Ref				
Tertiary	2.97(1.85–5.82)^a^	2.23(1.44–3.27)^a^	2.81(1.77–4.82)^a^	-	-
Secondary	3.70(1.97–7.64)^a^	2.64(1.81–3.84)^a^	3.29(1.95–5.63)^a^	-	-
Professional title					
Primary	Ref				
Intermediate	-	-	1.16(0.91–1.38)^b^	1.21(0.95–1.46)^b^	-
Senior	-	-	1.37(1.02–2.03)^a^	2.28(1.66–2.95)^a^	-
Weekly working hours					
< 40 h	Ref				
40 ~ 50 h	-	1.18(1.04–1.41)^b^	-	-	-
> 50 h	-	1.71(1.37–2.12)^a^	-	-	-

PA, physical assault; EA, emotional abuse; T, threat; VSH, verbal sexual harassment; SA, sexual abuse.“Ref” refers to the reference category.^a^*p* < 0.01.^b^*p* < 0.05. “-” indicates *p* > 0.05.

## 4 Discussion

WPV remains a significant problem in medical settings (2–4, 27). The present study investigated the factors associated with WPV against Chinese HCWs, while also examining the relationship between WPV and job satisfaction. Among our study population, the prevalence of WPV against HCWs was found to be 58.2%, with the highest incidence observed for EA at 49.7%. However, it is important to note that reported incidences of exposure to WPV can differ across nations. Previous research has reported rates of 75, 54, 48, and 45% among HCWs in India, Turkey, Saudi Arabia, and Italy, respectively, (3, 28–30). In a recent comprehensive analysis consisting of 17 studies, it was found that the prevalence of WPV among HCWs was estimated to be 47% (27). It is evident that cultural and geographic factors, as well as differences in perceptions and definitions of WPV, work schedules, occupations, study locations, and methodological approaches may contribute to variations in reporting frequencies of WPV. Furthermore, it is worth noting that attacks on HCWs have significantly increased in certain countries, such as the United States, Pakistan, and India, during the COVID-19 pandemic (9, 31). The implementation of infection control measures, such as quarantine and isolation, aimed at managing and preventing the dissemination of SARS-CoV-2, has unfortunately heightened the potential for patients and their families to engage in threatening or violent conduct (32).

Our findings revealed a greater occurrence of WPV among male HCWs compared to their female counterparts. This observation aligns with previous studies conducted in this field (18, 33). Furthermore, our study also identified the impact of working hours, work schedule (shift or non-shift work), and professional title on the likelihood of encountering WPV incidents. These results align with the existing literature (34–36). The influence of shift work on the occurrence of different forms of WPV can be explained by multiple factors such as understaffing during night shifts, staff fatigue, and subsequent impact on patient satisfaction (2, 37). In our study, HCWs holding senior professional titles reported a higher incidence of VSH and T compared to those with primary titles. In China’s healthcare system, the 3-tier responsibility framework for medical practitioners and nurses involves allocating greater responsibilities and a more demanding workload to those holding senior professional titles (18, 38). Patients often seek medical attention from higher-level professionals even for minor, self-limiting conditions. Consequently, the surge in patient load increases work pressure on physicians, leading to chronic overwork which may result in haste, indifference, and disrespectful behaviors toward patients (39). This phenomenon significantly contributes to doctor-patient tension (40). Furthermore, research has indicated that patients often hold elevated expectations toward doctors with senior professional titles (38). The inability to meet these expectations is a major contributor to incidents of assault (19). Our study also revealed that HCWs who work over 50 h per week had a 1.71-fold higher likelihood of experiencing EA in comparison to those working less than 40 h per week. This can be attributed to elevated levels of work-related stress and fatigue associated with longer working hours (41). These factors increase the risk of conflicts and violent behaviors during interactions with patients and their families (2). Moreover, extended working hours deprive HCWs of adequate rest and relaxation time, negatively impacting their mental and emotional well-being (42). This, in turn, affects their ability to deliver high-quality care.

In our study, we found that HCWs employed in secondary- and tertiary-level medical facilities encounter an elevated susceptibility to all five categories of WPV when contrasted with their counterparts in primary-level hospitals. This observation raises two potential explanations for this trend. Firstly, secondary- and tertiary-level hospitals often deal with patients who have more severe illnesses, which may increase the likelihood of encountering violent incidents (19). A previous study conducted in Thailand indicated that the severity of a patient’s illness plays a significant role in instigating physical violence initiated by the patient (43). Moreover, it has been highlighted in a Chinese study that unreasonable patient expectations can also lead to aggressive behavior from patients (44). Therefore, besides adequately explaining medical conditions to patients, addressing unrealistic expectations should also be considered to effectively reduce WPV in higher-level healthcare facilities. Secondly, top tier hospitals often concentrate highly educated healthcare professionals and have advanced medical equipment (39). As a result, primary care facilities, which are relatively underutilized and less competitive, may struggle to gain patient trust (45). Many patients even seek treatment for minor ailments at the outpatient services of secondary and tertiary hospitals (46). This leads to shorter consultation times per patient, potentially compromising the quality of care provided and decreasing patient satisfaction (47). To address the underlying factors contributing to WPV, it is crucial to improve the quality and utilization of primary care services as a long-term strategy within the healthcare system (19, 48).

One strength of our study is the identification of factors associated with different types of WPV. As mentioned earlier, common factors across all types of WPV include male gender, shift work, and employment at secondary and tertiary hospitals. EA is correlated with working over 50 h per week, while T and VSH are associated with holding senior professional titles. It is worth noting that educational attainment is correlated with PA, EA, and SA. HCWs with a bachelor’s or associate’s degree have a higher risk of experiencing PA, EA, and SA compared to those with a master’s degree or above. This disparity can be attributed to multiple factors (21, 32). First, HCWs with higher education qualifications, such as a master’s degree, are more likely to hold positions that involve less direct patient care or interaction. Those with a bachelor’s degree may be more frequently engaged in frontline work, where they face a higher risk of violence and abuse (21). Second, higher education qualifications are often associated with increased training in communication skills, conflict resolution, and coping strategies under stressful situations (20). HCWs with a master’s degree may possess better skills in managing conflicts and handling volatile situations, thereby reducing their susceptibility to WPV.

Our results revealed that as the level of violence increased, HCWs displayed a decreased inclination to remain in the healthcare industry and showed reluctance to encourage their children to pursue careers as healthcare professionals. Previous studies have also confirmed that experiencing high levels of WPV can have a negative impact on HCWs’ perceptions of their profession (3, 6). The increased exposure to violence not only affects their physical safety but also influences their emotional well-being and job satisfaction (6). The fear and stress generated by WPV may lead HCWs to question their career choices and discourage them from recommending the profession to their children due to concerns about safety and the potential negative impact on their personal and family lives (49). The absence of public condemnation toward the perpetrators in the wake of the news about doctors being killed due to WPV has exacerbated this situation (5). These phenomena highlight the pressing need for effective measures to prevent and address WPV in order to preserve the motivation, satisfaction, and retention of healthcare professionals, as well as to attract future generations to join the healthcare workforce (50, 51).

Efforts to reduce the escalating violence against HCWs necessitate a comprehensive approach (2). Merely advocating for harsher penalties, as endorsed by HCWs globally, is unlikely to suffice (4, 10). Instead, the following strategies can be implemented effectively to address this issue. First, addressing the persistent problem of staff shortages in public hospitals across China is paramount (39, 50). Allocating additional funding for the recruitment of more doctors and nurses will help alleviate the strain. This, in turn, will allow for longer patient consultations, particularly in overstretched public hospitals, enabling doctors to establish meaningful connections with their patients (39). Second, healthcare organizations should fully support HCWs who report incidents of verbal or physical violence (1, 50). This proactive approach will combat the issue of underreporting WPV, fostering a safer work environment. Third, organizing training programs for HCWs to recognize early signs of potential violence, manage hazardous situations, and ensure self-protection is essential (5). Equipping HCWs with these skills will bolster their ability to respond effectively to violent incidents. Fourth, timely communication with patients and their families regarding service delays, especially when certain conditions require prioritization, is vital (50). Finally, the media should responsibly promote trust and understanding between the general public and HCWs (2). Biased reporting and sensationalizing negative occurrences could escalate tensions and erode public trust. Media outlets should verify information before dissemination, considering the repercussions on HCWs’ reputation and the overall healthcare system.

## 5 Limitations

This study has several limitations. First, it is crucial to emphasize that the data utilized in this research were gathered retrospectively. Hence, there exists a dependence on the participants’ capacity to precisely recollect occurrences that took place within the preceding 12-month period. This introduces the possibility of recall bias and may impact the validity of the findings. Second, the study’s cross-sectional design hinders our ability to establish a definitive cause-and-effect relationship between variables. Third, it is worth noting that the e-survey tool utilized in this research aimed at engaging HCWs across diverse sectors and a variety of healthcare settings. However, due to the inability to control the distribution of the survey, there may be unequal representation across different sectors. Consequently, there is a possibility that the collected sample may not provide a comprehensive representation of the present situation of WPV among HCWs in China, limiting the generalizability of the prevalence measures reported in this study. Furthermore, it is important to consider the potential bias introduced by participants who provided responses to the online survey. These individuals may already be experiencing heightened stress levels due to their vulnerability to WPV, potentially influencing their responses. Despite these limitations, this study has identified crucial risk factors for WPV that have the potential to alleviate the occurrence of WPV among HCWs in China. In future research, it would be beneficial to delve deeper into the long-term effects of WPV on the mental and physical health of HCWs in China. Additionally, conducting longitudinal studies to examine the trends of WPV over time and assess the effectiveness of implemented interventions would provide further insights into addressing this critical issue.

## 6 Conclusion

The high prevalence of WPV against HCWs in China continues to be a cause for serious concern. Our study identified several independent factors associated with WPV, including male gender, engaging in shift work, possessing a bachelor’s degree, having a senior professional position, and working over 50 h per week. Additionally, we found that HCWs stationed at higher level care facilities, specifically secondary hospitals, confront a notably elevated probability of encountering WPV. This finding underscores the urgent need for increased focus on addressing and mitigating WPV occurrences within secondary hospitals. Furthermore, our findings demonstrated a strong association between increased levels of WPV and the heightened dissatisfaction experienced by HCWs in their chosen profession. In conclusion, our study underscores the importance of evaluating violence against HCWs as a key step in the development of effective measures to combat WPV and mitigate its adverse consequences.

## Data availability statement

The original contributions presented in the study are included in the article/Supplementary material, further inquiries can be directed to the corresponding author.

## Ethics statement

The studies involving humans were approved by the Ethics Committee of the Fourth People’s Hospital of Chengdu. The studies were conducted in accordance with the local legislation and institutional requirements. Written informed consent for participation was not required from the participants or the participants’ legal guardians/next of kin in accordance with the national legislation and institutional requirements.

## Author contributions

XY: Data curation, Investigation, Writing – original draft. T-tC: Investigation, Writing – original draft. S-yZ: Writing – review & editing. C-yL: Investigation, Writing – review & editing. LZ: Data curation, Writing – review & editing.
