# Supplementary material for: Factors associated with workplace violence against Chinese healthcare workers: an online cross-sectional survey
Source: Front Public Health. 2024 Mar 14;12:1295975. doi: 10.3389/fpubh.2024.1295975 (PMC10977601; doi:10.3389/fpubh.2024.1295975)
Supplement: Supplementary file 1 [file Data_Sheet_1.doc]

**Additional file 1:** Details of the questionnaire in the study.

**1. Demographic information**

1. Your Gender:

□ Female

□ Male

1. Your Age:
2. Your Marital Status:

□ Unmarried

□ Married

1. Your level of education:

□ Associate’s degree or below

□ Bachelor’s degree

□ Master’s degree or above

**2. Professional information**

1. The level of the hospital in which you are employed:

□ Primary

□ Secondary

□ Tertiary

1. In which department are you employed at the hospital?
2. Are you a doctor or a nurse?

□ Doctor

□ Nurse

1. Are you engaged in shift work or not?

□ Shift work

□ Non-shift work

1. What is your current professional title?

□ Primary

□ Intermediate

□ Senior

1. How many hours do you work in a week? Hours
2. How many years have you worked as a medical worker? Years
3. **Workplace Violence Scale**

**Over the past year**, have you experienced any of the following forms of violence initiated by patients or their family members? After familiarizing yourself with the definition of each type of violence, please select the corresponding frequency from the provided options.

| **five types of violence** | **Times encountered in the past year** | | | |
| --- | --- | --- | --- | --- |
|  | None | 1time | 2-3times | ≥4times |
| 1. Physical assault (pushing, biting, beating, spitting) |  |  |  |  |
| 1. Emotional abuse (cursing, disrespect and disparagement words) |  |  |  |  |
| 1. Threats (use of verbal, written or physical force resulting in fear of negative consequences) |  |  |  |  |
| 1. Verbal sexual harassment (unwelcome remarks or comments of a sexual nature) |  |  |  |  |
| 1. Sexual abuse (unwanted touching or other sexual behaviors) |  |  |  |  |

1. **Career satisfaction questions**
2. Given the opportunity, would you still opt for a career in medicine?

□ Yes

□ No

1. Do you wish for your child to become a healthcare professional?

□ Yes

□ No
